# Navigating the Evolving Landscape of COVID-19: Strategies to Increase Vaccine Confidence and Improve Vaccination Rates in the United States

**DOI:** 10.3390/vaccines12091072

**Published:** 2024-09-19

**Authors:** James A. Mansi, Heather R. Hensler, Rachel Dawson, Reed Tuckson, Todd Wolynn

**Affiliations:** 1 Moderna, Inc., Cambridge, MA 02142, USA; 2 Black Coalition Against COVID, Atlanta, GA 30328, USA; 3 Kids Plus Pediatrics, Pittsburgh, PA 16046, USA; 4 Shots Heard Round the World, Pittsburgh, PA 16046, USA

**Keywords:** COVID-19 vaccine, 2023–2024 COVID-19 vaccine, COVID-19 vaccine confidence, raising COVID-19 vaccination rates

## Abstract

The COVID-19 pandemic has had a significant impact on every individual in the United States. The launch of the COVID-19 vaccines is estimated to have averted millions of deaths and reduced over 18 million COVID-19-related hospitalizations. In September 2023, the updated 2023–2024 COVID-19 vaccine, which includes a monovalent component that corresponds to the omicron variant XBB.1.5, reflecting the predominant circulating variant at the time of strain selection, was approved and was recommended for use in all people ≥ 6 months of age. Despite this recommendation, the US uptake of the updated COVID-19 vaccines over the 2023–2024 season has been far from optimal, placing many people at unnecessary risk of severe COVID-19 outcomes. This paper provides an overview of the current state of COVID-19 in 2023–2024 and barriers to vaccine uptake. With the continued evolution of the virus, the potential for more virulent variants, reduced public acceptance of vaccination, and the potential barriers that contributed to low vaccine uptake are explored to provide solutions for improving COVID-19 protection for future seasons.

## 1. Introduction

The COVID-19 pandemic has evolved significantly since the first case was reported in Wuhan, China in December 2019. From the start of the pandemic to 6 April 2024, the CDC reports that there have been close to 1.2 million COVID-related deaths and 7 million hospitalizations in the United States [1]. A large number of these COVID-19-related deaths occurred prior to the implementation of a universal immunization program in the United States [2]. Notwithstanding, there remains a significant health burden related to COVID-19, and it remains the leading respiratory infectious disease responsible for hospitalizations and deaths regardless of age or comorbid status.

Vaccines have changed the trajectory of the pandemic. It has been estimated that between 12 December 2020 and 30 November 2022, COVID-19 vaccines have averted over 3.2 million COVID-19 deaths, 18.5 million COVID-19 hospitalizations, and close to 120 million COVID-19 infections in the US [3]. The public desire for COVID-19 vaccines was very high when introduced, with an estimated 80.6% of all American adults (≥18 years) having received at least one dose [4]. As the virus has continued to evolve, additional doses and new variant-specific formulations designed to protect the population from the rapidly evolving SARS-CoV-2 virus have been introduced. The uptake of subsequent vaccine doses has been significantly lower than the first two doses.

The Public Health Emergency (PHE) was rescinded on 10 May 2023. Although COVID-19 is no longer considered a public health emergency, it continues to be the leading respiratory infectious disease and remains a persistent public health threat. This is due in part to the emergence of variants with enhanced transmissibility and pathogenicity. A new vaccine was approved on 11 September 2023, targeting the most prominent variant at the time of selection, XBB.1.5. This was followed by a simple recommendation from the Advisory Committee on Immunization Practices (ACIP) on 12 September 2023 that every person 6 months of age and older receive at least one dose of the 2023/24 COVID-19 vaccine. Even with this clear and simple recommendation, as of 31 March 2024, only 22.6% of eligible adults and 14% of children (6 months to 17 years) hadreceived it [4,5]. This low vaccine uptake occurred during a period where there were approximately 1000–2500 COVID-19 deaths and 6500–30,000 hospitalizations due to COVID-19 each week [6].

With the ongoing impact of COVID-19 and the high likelihood of a new variant with higher virulence or an immune escape mechanism, it is important for governments, public health officials, and healthcare providers to learn from some of the challenges of 2023–2024 fall/winter season to develop strategies to improve vaccine confidence and uptake. This could raise COVID-19 vaccination rates, thus improving patient outcomes and supporting public health objectives. In this paper, we will review the key lessons learned from the 2023–2024 season and provide some potential strategies to address key barriers to COVID-19 for future seasons.

## 2. COVID-19 Vaccine Approval and Delivery within the United States

The approval, distribution, and delivery of COVID-19 vaccines are highly regulated within the United States. The regulatory approval and recommendations for vaccines occur through several committees. The Vaccines and Related Biological Products Advisory Committee (VRBPAC) reviews and evaluates data concerning the safety, effectiveness, and appropriate use of vaccines and related biological products that are intended for use in the prevention, treatment, or diagnosis of human diseases [7]. They make appropriate recommendations to the Commissioner of Food and Drugs on the approval of vaccines [7]. Once a vaccine is approved by the FDA, the Advisory Committee on Immunization Practices (ACIP) develops recommendations on how to use vaccines to control disease in the United States [8]. The Committee’s recommendations are forwarded to the Center of Disease Control’s (CDC’s) Director and become official CDC policy once adopted [8].

After a vaccine is recommended by the CDC, the manufacturers of the vaccine work with a variety of partners to support healthcare providers in delivering vaccines to recommended recipients.

## 3. COVID-19 During the 2023–2024 Season

Although the US public health emergency for COVID-19 ended on 11 May 2023, it continues to be a leading cause of hospitalizations due to respiratory infections. Between 2 September 2023 and 31 March 2024, there were over 600,000 hospitalizations and over 45,000 deaths due to COVID-19 in the US [2]. At its peak, there were approximately 35,000 new hospitalizations and 2500 deaths per week. Like influenza, COVID-19 activity, hospitalizations, and deaths tend to follow peaks during the traditional winter viral respiratory season. However, unlike influenza, which typically drops below endemic levels (inter-seasonal levels), COVID-19 is responsible for significant burdens throughout the year. Whilst the burden of disease has varied between the different age groups, morbidity and mortality remained significant regardless of age (Table 1), supporting the CDC recommendations that all persons aged 6 months and older should receive the updated 2023–2024 COVID-19 vaccine [9].

### 3.1. Pediatric Population

COVID-19 infection and illness continue to be a serious and urgent concern as hospitalization rates from 2020–2024 have consistently been high between October and January [2]. Children who were hospitalized had low vaccination rates. Only 4.5% of children hospitalized due to COVID-19 completed their primary series against COVID-19 and 7% of children hospitalized initiated but did not complete their COVID-19 primary vaccination series [12]. Unlike what is seen in adults, almost half of children hospitalized for COVID-19 had no underlying conditions [12].

### 3.2. Adults Aged 18–49 Years

Although this group was once thought to have a low risk of COVID-19 complications, they are still at risk of hospitalization. The rate of COVID-19 hospitalizations in adults aged 18–49 was 37.4 per 100,000 people during the 2023–2024 season [10]. Low vaccination rates in this age group could affect hospitalization risk. During the 2022–2023 season, 42% of those hospitalized due to COVID-19 aged 18–49 were unvaccinated and only 8% of those hospitalized had received the 2022-2023 bivalent COVID-19 vaccine [13].

### 3.3. Adults Aged 50–64 Years

With increasing age, there is a higher COVID-19 hospitalization and comorbidity risk. The cumulative hospitalization rate for the 2023–2024 season was 109.2 per 100,000 people [10]. Like in other age groups, the hospitalization risk was much higher in those who were not up to date with the COVID-19 vaccine recommendations. During the 2022–2023 season, 26% of those hospitalized due to COVID-19 aged 50–64 years were unvaccinated and only 13% of those hospitalized had received the 2022–2023 bivalent COVID-19 vaccine [14].

Comorbidities are higher in this age group. Indeed, 69.5% of adults aged 55–64 have at least one chronic condition. This age group was not included in ACIP’s February 2024 COVID-19 additional dose recommendation, [15]. Compared with those aged 18–29 years, the risk of death is 25 times higher in those aged 50–64 years [14].

### 3.4. Older Adults Aged ≥ 65 Years

As was seen in the first years of the pandemic, this age group was the most impacted by severe outcomes. Compared with those aged 18–29 years, the rate was 60 times higher in those aged 65–74 years, 140 times higher in those aged 75–84 years, and 340 times higher in those aged ≥ 85 years [16]. The cumulative hospitalization rate for the 2023–2024 season was 537.6 per 100,000 people [14]. Comparatively, adults aged 65 years of age and older represented 218.2 per 100,000 people hospitalized due to influenza during the 2023–2024 season [11]. Nearly one in six hospitalized adults over 65 years of age were residents of long-term care facilities [17]. During the 2022–2023 season, 17% of those hospitalized due to COVID-19 aged 65 years of age and older were unvaccinated and only 25% of those hospitalized had received the 2022-2023 bivalent COVID-19 vaccine [13].

Comparatively, older adults represented 52% of all influenza-related hospitalizations during the 2022–23 influenza season [18]. As was seen with other age groups, the hospitalization burden was higher in those who were not up to date with current vaccine recommendations.

### 3.5. Risk Factors for Severe COVID-19 Outcomes

**Figure 1:**
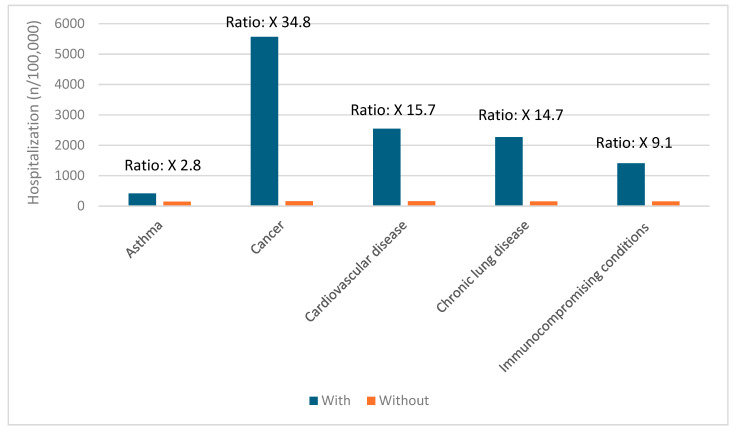
Risk of hospitalization due to COVID-19 for ages 0–5 years based on common comorbidities (Veradigm dataset April 2024) [22,23].

**Figure 2:**
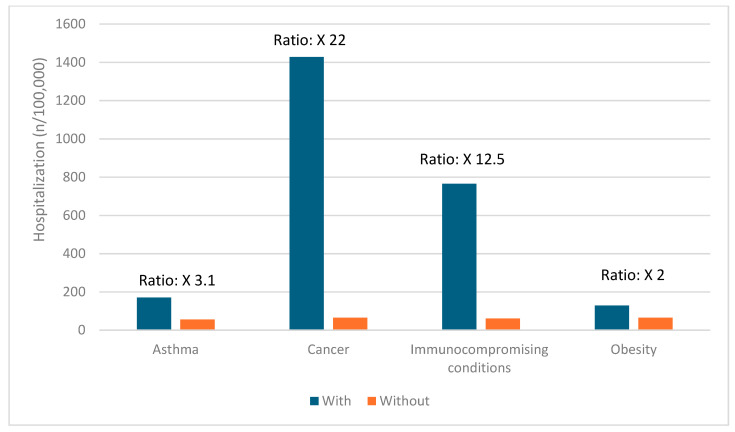
Risk of hospitalization due to COVID-19 for ages 6–17 years based on common comorbidities (Veradigm dataset April 2024) [22,23].

**Figure 3:**
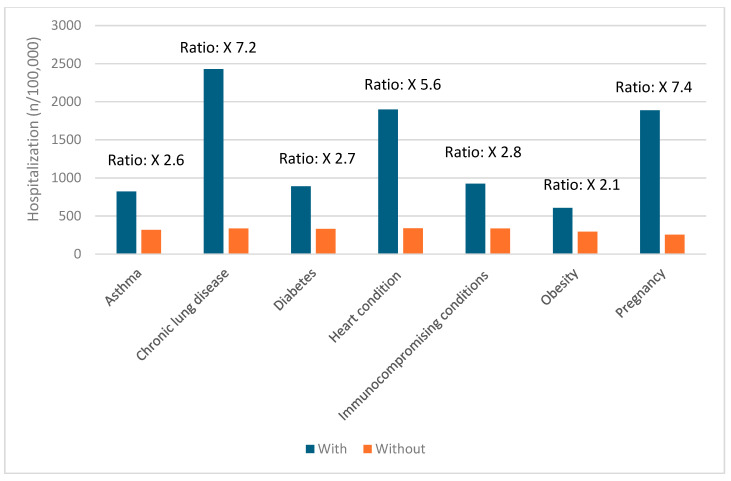
Risk of hospitalization due to COVID-19 for ages 18–49 years based on common comorbidities (Veradigm dataset April 2024) [22,23].

**Figure 4:**
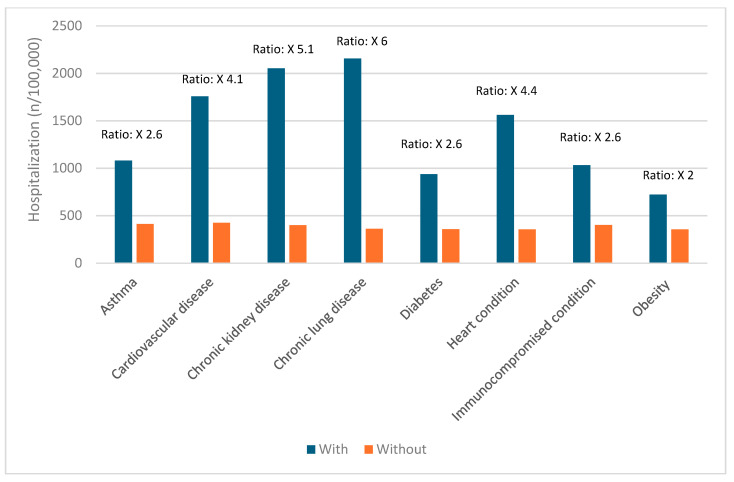
Risk of hospitalization due to COVID-19 for ages 50–64 years based on common comorbidities (Veradigm dataset April 2024) [22,23].

**Figure 5:**
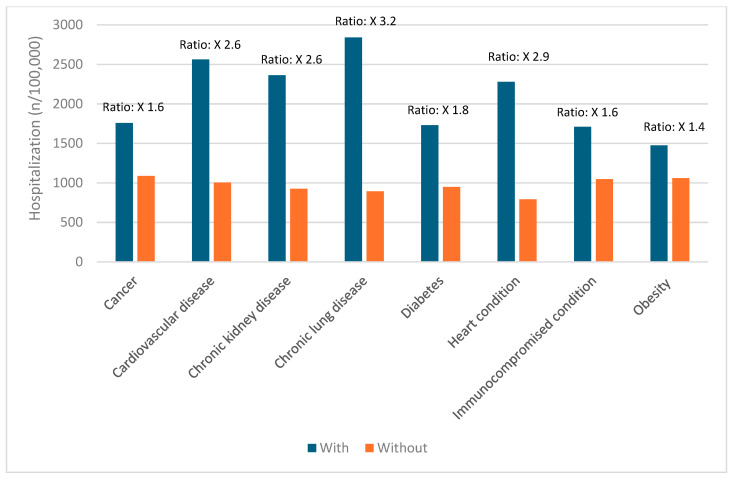
Risk of hospitalization due to COVID-19 for ages ≥ 65 years based on common comorbidities (Veradigm dataset April 2024) [22,23].

Certain groups continue to be at higher risk of severe COVID-19 outcomes across all ages [19]. These include infants, older adults, and people with underlying medical conditions or certain disabilities (Figure 1, Figure 2, Figure 3, Figure 4 and Figure 5) [19]. During the first seven months of 2023, older adults (≥65 years) accounted for 63% of hospitalizations and 88% of in-hospital COVID-19-related deaths [19]. Of this group, 90% of those hospitalized had multiple pre-existing medical conditions [19]. It is estimated that almost one in two US adults have at least one comorbid condition that increases the risk of COVID-19 complications [20]. Many of these adults have multiple comorbidities, further increasing the risk of COVID-19 [21]. As the number of comorbidities increases, the risk of death, invasive mechanical ventilation, and ICU admission also increases [21].

### 3.6. The Compounding Effect of Long COVID

Beyond hospitalizations and deaths, COVID-19 has been associated with an increased risk of long COVID. The impact of long COVID on the affected patient and the population is significant and tends to impact a different population than those at risk of COVID-19-related hospitalizations [24].

It is estimated that 5.3% of US adults or 13 million adults are currently experiencing long COVID as of 1 April 2024 and 3 million Americans are living with a disabling condition due to long COVID [24]. Based on 2020 US census population data, this would translate to approximately 46 million adults who have experienced long COVID [25]. The highest reported prevalence of long COVID was seen in people aged 40–49 years [24]. The full impact of COVID-19 in this age group extends beyond hospitalizations.

Long COVID may also affect children, irrespective of whether they are at risk or healthy. The prevalence of long COVID in children was estimated at 23.4% in a meta-analysis of 40 studies including over 12,424 children [26]. The prevalence of any symptoms during 3–6, 6–12, and >12 months was 26.4%, 20.6%, and 14.9% [26].

**Figure 6:**
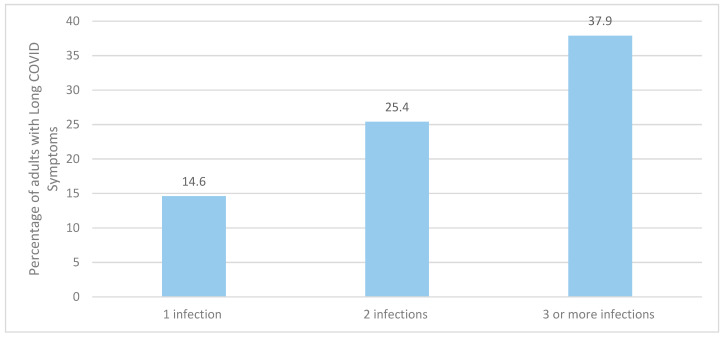
Percentage of adults with long-term symptoms, by number of self-reported COVID-19 infections [27].

The risk of long COVID is impacted each time the individual is infected with COVID-19. Reinfection further increases the risks of complications in the acute and post-acute phases versus those with no reinfection. The risk of at least one sequela is three times higher in those with a history of three or more infections (Figure 6).

Long COVID has a significant burden on the healthcare system, with significantly increased healthcare utilization and cost and an estimated cost for the US of USD 3.7 trillion through to 2022 [28]. The Household Pulse Survey by the US Census Bureau states that 2–4 million Americans are out of work due to Long COVID with an estimated cost of those lost wages of around USD 170 billion a year [24]. It is estimated that approximately 5.3% of US adults (~13.6 million adults) are currently experiencing long COVID symptoms [24]. One study found that the cognitive deficits associated with long COVID in those hospitalized for COVID-19 were equivalent in magnitude to 20 years of aging [29]. It is estimated that close to one in four American adults who currently have long COVID have significant activity limitations [24].

### 3.7. COVID-19 Burden vs. Influenza

COVID-19 continues to cause a more significant burden than influenza in the US. Through the 2023–2024 season, the cumulative rate of hospitalization due to COVID-19 was higher than for influenza during the same time period [30,31]. This was also consistent with the 2022–2023 season (Table 2) [23]. Roughly 12% of hospitalizations with either COVID-19 or influenza included ICU admission [23].

The hospitalization risk for COVID-19 was higher than influenza in all age groups. This difference in impact was especially seen in older adults (≥65 years), where the number of COVID-19-related hospitalizations was more than double that seen with influenza [30]. Although the impact of influenza is less than seen with COVID-19, the immunization rate for influenza in US adults (≥18 years), as of 31 March 2024, was more than double that of COVID-19 (48.1% vs. 22.6%) [4,32].

### 3.8. The Continued Evolution of COVID-19

The SARS-CoV-2 virus has continued to evolve through the fall and winter of 2023/24 [33]. During the summer and early fall of 2023, omicron XBB variants were co-circulating with no clear dominance [33]. Through the winter of 2024, the JN.1 variant became the predominant circulating variant and continued into the spring [33]. The emergence of the JN.1 variant coincided with an initial increase in hospitalizations due to COVID-19 [2]. As of 25 May 2024, KP.2 became the predominant circulating variant (28.5%), with JN.1 representing only 8.4% of the circulating variants [33]. The continued evolution of the SARS-CoV-2 virus could have a significant impact on the entire US healthcare system, such as hospitalizations in people with waning immunity. COVID-19 has not settled into a seasonal pattern, and it is impossible to predict which variant will be next. Owing to the fact that the SARS-CoV-2 virus continues to mutate, vaccines are updated to target the predominating variant.

## 4. COVID-19 2023/2024 Vaccine

### 4.1. FDA Approval and ACIP Recommendation

On 11 September 2023, the Food and Drug Administration (FDA) approved the updated (2023–2024 formula) COVID-19 mRNA vaccines by Moderna and Pfizer-BioNTech for persons aged ≥ 12 years and authorized these vaccines for persons aged 6 months–11 years under Emergency Use Authorization (EUA) [9]. The updated COVID-19 vaccines included a monovalent XBB.1.5 component, which targeted the circulating variants at that time [9]. On 12 September 2023, ACIP recommended vaccination with the updated (2023–2024 Formula) COVID-19 vaccine for all persons aged ≥ 6 months [9].

### 4.2. Safety, Immunogenicity, and Effectiveness of the Vaccine

The immunogenicity of the 2023/2024 vaccine formulation significantly increases the virus-neutralizing antibodies for a variety of circulating variants [34]. In uninfected individuals, the vaccine boosted serum virus-neutralization antibodies significantly against not only XBB.1.5 (27.0-fold) but also the other variants EG.5.1, HV.1, HK.3, JD.1.1, and JN.1 (13.3- to 27.6-fold) [34]. The antibody response in individuals infected by the Omicron subvariant was significantly higher (1504- to 22,978-fold higher) than in the uninfected group [34]. Studies evaluating safety did not find an increase in new safety signals and most adverse events were mild to moderate in severity [35,36]. A nationwide cohort study of more than 1 million older adults (≥65 years) receiving the new vaccine formulation found no increase in 28 adverse events following administration [37].

The vaccine effectiveness (VE) of the 2023/2024 vaccine was evaluated across two large CDC networks (VISION and IVY) [38]. VE estimates against COVID-19-associated hospitalization were 52% (95% CI = 47–57%) and 43% (95% CI = 27–56%) [38]. Another study evaluated the effectiveness of Moderna’s Omicron XBB.1.5 vaccine (mRNA-1273.815) at preventing COVID-19-related hospitalizations and any medically attended COVID-19 in adults ≥ 18 years, both overall and by age and underlying medical conditions [39]. Overall, 859,335 matched pairs of mRNA from 1273.815 recipients and unexposed adults were identified in the large electronic health record database [39]. Among the overall adult population (≥18 years), VE was 60.2% (53.4–66.0%) against COVID-19-related hospitalization and 33.1% (30.2–35.9%) against medically attended COVID-19 over a median follow-up of 63 (IQR: 44–78) days [39].

On 27 June 2024, the ACIP recommended the 2024–2025 COVID-19 vaccine for all individuals ≥ 6 months of age [40]. ACIP assessed the value of a universal recommendation for the 2024–2025 COVID-19 vaccine versus a recommendation for only high-risk individuals [40]. Based on their modeling of vaccine effectiveness, a universal vaccine recommendation for all Americans ≥ 6 months of age would lead to a reduction in approximately 30,000 hospitalizations over the next year in the U.S. compared to only immunizing high-risk individuals [40].

COVID-19 vaccines have a favorable safety profile as demonstrated by robust safety surveillance over 3 years of COVID-19 vaccine use [41]. Anaphylactic reactions have been rarely reported following receipt of COVID-19 vaccines [41]. A rare risk of myocarditis and pericarditis has occurred; however, this is predominately in males aged 12–39 years [41]. No new safety concerns had been identified for the 2023–2024 Formula COVID-19 vaccine. Local and systemic reactions are commonly reported following the administration of COVID-19 vaccines and this was seen more commonly with adolescents and younger adults compared to older adults [41].

During the COVID-19 pandemic, higher mortality rates and shorter life expectancy have been reported in Japan and many other countries [42,43,44,45,46,47]. A safety study was conducted within the United States evaluating non-COVID-19 mortality risk following COVID-19 vaccination to determine if there was any potential causal link [48]. The authors evaluated the Vaccine Safety Datalink (VSD) sites, which encompass over six million vaccine recipients [48]. After adjustment for extensive confounders, the authors concluded that: [48]

- Recipients of BNT162b2, mRNA-1273, and Ad26.COV2.S vaccines had lower non-COVID-19 mortality risk than their comparator groups.
- The findings suggested some all-cause mortality benefits of COVID-19 vaccines for unknown causes in addition to their known protection against COVID-19 infection, severity of the disease, and death.
- No increased risk was found for non-COVID-19 mortality among recipients of COVID-19 vaccines used in the US.

### 4.3. 2023/2024 Vaccine Uptake in Different Populations

As mentioned, the uptake of the 2023/2024 COVID-19 vaccine was far from optimal. Across all age categories, it was lower than the 2023/2024 influenza vaccine (Table 3). The uptake of the 2023/2024 vaccine was also lower in all minority US adults (Asian, Black, Hispanic, multiple races, Pacific Islander, American Indian) compared to White, non-Hispanic US adults (Table 4) [4]. This reflects a decline from the vaccination rates reported in January 2022 where, thanks to efforts from partnerships across community-based organizations and black clergy, the gap between black communities was closed. During this period, black life expectancy in the second year was better than white Americans. This further highlights the importance of community engagement coupled with supporting resources and funding to effectively close the vaccination gaps and raise life expectancy.

### 4.4. Attitudes and Intentions for COVID-19 Vaccination

Attitudes and intentions regarding COVID-19 vaccination provide insight into COVID-19 immunization for future seasons. The Omnibus survey conducted from 5 to 29 January 2024, in adults ≥ 18 years of age, asks about concerns and intentions regarding COVID-19 vaccination. Of those responding that they would probably or definitely not get the COVID-19 vaccine, the concerns raised were predominantly around safety, with 47% stating unknown serious side effects and 33.2% having concerns about heart-related issues. Trust was another leading concern, with 39% of the respondents indicating that there were not enough human studies on the COVID-19 vaccine or that they did not trust the government or pharma (37.2%). The effectiveness of the COVID-19 vaccine was cited by 32.9% of the respondents.

Not surprisingly, concerns leading to not receiving the COVID-19 vaccine can be grouped into three main categories, notably:

- Perceived risk of getting ill or severity of COVID-19
- Effectiveness of the COVID-19 vaccine.
- Safety of the COVID-19 vaccine.

Although there was a sizeable group of individuals who responded that they probably or definitely would not get the COVID-19 vaccine because they do not trust the government or the pharma industry (37.2%), there remains a significant body of evidence indicating that patients and parents view their HCP as the most trusted source [49].

## 5. Adapting Strategies for Future COVID-19 Seasons

The SARS-CoV-2 virus will likely remain endemic in the US and worldwide and seasonality compared to other respiratory viruses (e.g., influenza and RSV) has not been established. Ongoing strategies will be required to protect the US population and achieve public health goals. The 2023/2024 season highlighted a need for adjustments in the delivery of COVID-19 vaccines as significant gaps have been identified. Three key areas that can address many of the issues are:

- Changes in the COVID-19 vaccine approval process to align with influenza to allow for co-administration.
- Increased integration of the COVID-19 vaccine into routine delivery of care.
- Communication and engagement with healthcare providers for optimal patient education, overcoming the challenges of misinformation and distrust, along with vaccine delivery.

### 5.1. COVID-19 Vaccine Approval Process

**Figure 7:**
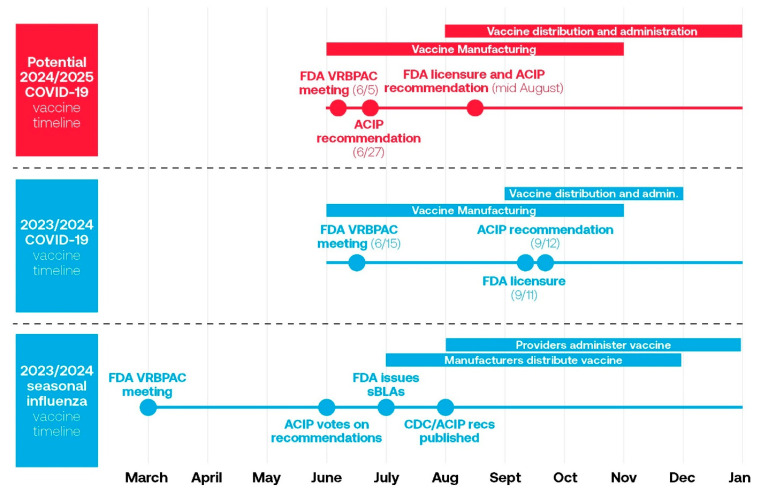
2023/2024 seasonal influenza vaccine timeline and 2023/2024 and potential 2024/2025 COVID-19 vaccine timeline [50]. ACIP, Advisory Committee on Immunization Practices; FDA, Food and Drug Administration; VRBPAC, Vaccines and Related Biologic Products Advisory Committee.

The timing of the approval of the 2023/2024 COVID-19 vaccine may have contributed to the lower vaccine uptake in the United States. Although it would be ideal for the delivery of the influenza and 2023/2024 COVID-19 vaccines to be at the same time, there was a difference in their approval timings [50]. The fall influenza vaccine approval was initiated in March 2023 with strain selection, with CDC/ACIP recommendations made in June 2023 (Figure 7) [50,51]. This allowed for the manufacturing and distribution of vaccines to allow providers to administer the vaccine prior to the influenza season. For the 2023/2024 COVID-19 vaccine, there was a delay in approval, FDA licensure, and ACIP recommendations. This significantly hampered vaccine distribution and uptake. This created challenges for the manufacturing and distribution of the vaccine in the early fall and resulted in COVID vaccines for the 2023/24 season becoming available 6–8 weeks after flu vaccines were available [50].

The proposed new timeline for the 2024/2025 COVID-19 vaccine would have the FDA VRBPAC meeting in June 2024 for strain selection, with ACIP recommendations in June versus September [50]. This allows for earlier file submission and approval (before September), vaccine manufacturing, and distribution with administration to occur similarly to the influenza vaccine. Fundamentally, the change in approval should help to simplify manufacturing, distribution, and communication, as well as alignment with influenza vaccine recommendations [50].

### 5.2. Increased Integration of the COVID-19 Vaccine into Routine Delivery of Care

The 2023/2024 COVID-19 vaccine uptake demonstrates a need for a change in the way the vaccine and its recommendations are communicated to everyone ≥ 6 months. Minorities have some of the lowest vaccine uptake rates. Vaccination coverage in January 2024 was highest for non-Hispanic white adults (24%) but lower for Asian adults (19.2%), Black adults (16.4%), Native Hawaiian or Other Pacific Islander adults (14%), Hispanic adults (13%), American Indians, and Alaska Native adults (11.4%) [52]. Moving forward, there is an imperative for public health to collaborate with any person and group that regularly comes into contact with the people at risk of COVID-19 to discuss and promote vaccination. Simplified and trusted vaccine messaging from trusted sources at the point of care or in the community can help to increase awareness and address vaccine confidence and complacency [53]. This message can be delivered by public health, community partners, and healthcare providers to improve vaccine awareness and uptake.

In the past, adult immunization was discussed in what was perceived as the fall ‘vaccine season’. This was primarily due to the only consistent respiratory adult vaccine being the fall influenza vaccine. The adult vaccine schedule is becoming more crowded. To meet immunization targets, it is important for all healthcare providers and public health to start and continue to have vaccine discussions outside of the fall and winter seasons [54]. Vaccines that are unlikely to require formula changes or those that offer a multiple-year duration of protection (e.g., pneumococcal and herpes zoster) could be administered outside of the fall season to allow for communication to be focused on influenza and COVID-19 during the peak respiratory virus season.

### 5.3. Engaging Providers for Fall 2024 Delivery

With the proposed update in the 2024/2025 COVID-19 vaccine approval process, there is an opportunity to engage public health, healthcare systems, and providers in the planning, distribution, and administration of the COVID-19 vaccine to the public. Having clear and early recommendations will allow healthcare systems and providers to have the appropriate supply and can reduce the wastage of the vaccine. By adapting the communication with providers to match that of influenza, there is a potential to increase the number of COVID-19 immunizations and enhance the knowledge of who should receive the 2024/2025 vaccine and when it should be administered.

The location of the administration of influenza and COVID-19 vaccines differs significantly. Pharmacies have administered almost every adult dose of the 2023/2024 COVID-19 vaccine (92.7% of all doses) [55]. This is not the case with the 2023/2024 adult influenza vaccine where administration location is more split between pharmacies (59.5% of doses) and medical offices (40.5% of doses) [56]. There was also a significant difference in the number of doses administered between the two vaccines, where in March 2024, the 2023/2024 COVID-19 vaccine administered was more than half that of influenza [55,56]. This disparity in where vaccines are being administered points to the need to engage more healthcare providers beyond pharmacies to take a more active role in COVID-19 vaccine administration.

With the differences in the vaccine uptake of influenza and COVID-19 vaccines, there should be an increased need to educate providers on the shared risk factors between these two infections. With the ACIP/CDC providing clear recommendations for the co-administration of the two vaccines, healthcare providers should be strongly encouraged to administer both vaccines on the same date for those ≥ 6 months of age [57]. The disconnect between the number of people receiving influenza versus COVID-19 vaccines indicates that if COVID-19 discussions were occurring at the point of care, there could be a higher vaccine uptake for future COVID-19 vaccines.

## 6. Addressing Key Barriers to COVID-19 Vaccine Uptake

With the low uptake of the 2023/2024 COVID-19 vaccine, there is a need for strategies that can help healthcare providers increase COVID-19 vaccine uptake. These include programs that can address vaccine complacency, convenience, and confidence. We propose this can be facilitated with a structured approach to improve vaccine access, simplifying vaccine messaging and the delivery of COVID-19 vaccines, and strategies that address misinformation and disinformation surrounding COVID-19 and its vaccines.

### 6.1. Improving Vaccine Access

The lack of COVID-19 vaccine access could be contributing to both vaccine complacency and convenience. When COVID-19 vaccines were first available, the majority of the public were willing to seek out vaccine locations and wait for the opportunity to receive a vaccine that could reduce their risk from this emerging infection. As the pandemic has progressed and public health measures have been rolled back, there is now a complacency amongst many individuals on the need for receiving additional COVID-19 doses. We must not lose touch that every healthcare provider, regardless of their area in research, clinical, academia, public health, and policy, are all touch points to enhance trust and overcome mistrust. They are an opportunity to provide evidence-based health guidance and overcome misinformation.

The last 4 years of the COVID-19 pandemic have demonstrated that the SARS-CoV-2 virus is unpredictable and will likely continue to evolve. Future variants may be associated with a higher risk of severe outcomes and long COVID, especially in those individuals who have missed recommended vaccine doses. To protect against future variants, there is a need to actively engage all groups (e.g., public health, primary care providers, pharmacists, community groups, and partners) and provide them with tools to facilitate a discussion on the potential impact of COVID-19 on their health and steps that can be taken to lower their risk. This helps to ensure all Americans have the information and access to make the decisions for mitigating their COVID-19 risk.

### 6.2. Simplifying Vaccine Messaging and the Delivery of COVID-19 Vaccines

The success of the public health vaccination program is contingent on a simple narrative on the role of vaccines in protecting the individual, their circle of contacts, and their community [58]. Vaccine messaging is crucial. Simply providing individuals with more vaccine and disease information was shown in one study to increase misperceptions or reduce vaccination intention [59].

Healthcare provider recommendations are strongly associated with vaccine uptake [60]. Individual doctors are the most trusted source, with 93% of the public saying they have a great deal or a fair amount of trust in their own doctor to make the right recommendations on health issues [49]. By making a strong recommendation to receive a vaccine, many patients are willing to accept it with little further discussion. Provider recommendations are especially effective if they take the form of a presumptive offer (Table 5) [61]. With this format, the default response is to accept vaccine uptake [60]. One study found that the use of presumptive language with respect to vaccine discussions significantly increased vaccine uptake and lowered vaccine resistance [62]. The advantage of this method for healthcare providers is that it has been shown to be time-sensitive and can simplify vaccine discussions with patients.

Occasionally, immunizers encounter a person who is hesitant to receive a vaccine despite a presumptive recommendation. It is important for healthcare providers to explore the reason(s) for hesitancy and engage the patient/caregiver in a discussion that could address their concern(s) [63]. As mentioned above, the vast majority of people who had concerns regarding the use of the COVID-19 vaccine were hesitant based on three categories:

- The perceived risk of getting ill or the severity of COVID-19
- Effectiveness of the vaccine.
- Safety of the vaccine.

Like with patients, there is some complacency regarding COVID-19 with healthcare providers. To provide them with tools and messages that can facilitate immunization for future COVID-19 seasons, an appropriate first step would be to link COVID-19 and influenza communication. This could stress the overlap in the high-risk groups and the importance of receiving both vaccines for optimal protection.

Another strategy is to engage trusted community messengers and support. This should be strongly encouraged to reach many different groups. These messengers play an important role in efforts to combat the proliferation of health misinformation.

### 6.3. Combating Misinformation and Disinformation

COVID-19 accelerated the spread of health information across borders, but studies have shown that accurate information does not spread as easily as misinformation [64]. Misinformation is information that is false, inaccurate, or misleading according to the best available evidence at the time [64]. It is the most common cause of false information and anyone can fall victim to it, including healthcare providers. Someone who spreads misinformation does not necessarily mean to cause harm, they simply believe the false information to be true. However, misinformation can still have real consequences when it comes to people’s health. Conversely, disinformation is false information that someone deliberately shares to deceive or manipulate others. Whilst misinformation could be spread innocently, people who spread disinformation do so knowing it is false.

Infodemiology studies the vast amount of health information that bombards us every day, helping to track the spread of information, identifying misinformation and disinformation, and helping to inform where and how to disseminate information. This type of intelligence is critical for healthcare providers. The misinformation that drives online conversations day after day is the misinformation that healthcare providers will hear from their patients when delivering a vaccination recommendation. To be better prepared for the current concerns or misconceptions impacting their patients’ healthcare decisions represents a lifeline connecting providers with their patients and the communities which they serve. This is critical given that a recent KFF survey found that 93% of patients and parents have a great deal of trust in their HCP to make the right recommendations on health issues. Healthcare providers are the first line of defense against infections and now misinformation.

Infodemiology organizes the information into dashboards that provide insights into public sentiment, trending topics, misinformation, gaps in knowledge, and potential opportunities for health interventions. These dashboards organize the cacophony of spiking misinformation topics and reactions which have helped to highlight persistent emerging themes. This is especially important for public health and healthcare providers as these themes point to the long-standing concerns of people they see in practice, for example, misinformation pertaining to having had COVID-19 in the past and that vaccination will not help protect them in the future. This would fall under the theme of perceived risk, which the provider can address succinctly and with evidence based on the patient’s age, medical history, and comorbidities. Similarly, people are less likely to discuss the safety of the vaccines than whether they would be forced to get vaccinated or carry vaccine verification. By focusing on individual posts, researchers are failing to see the larger picture: People are not influenced by one post so much as they are influenced by the narratives that these posts fit into.

## 7. Key Takeaways and Recommendations

Throughout this paper, we have highlighted the challenges of previous seasons and opportunities to address key barriers to immunization. Table 6 provides key recommendations for the government, public health officials, and healthcare providers to utilize to increase COVID-19 vaccine uptake in the future and protect the population against this ongoing threat. It is important to note that there have already been significant organizing efforts to bring together the whole health ecosystem to address vaccine uptake concerns, for example, the Coalition for Trust in Health & Science, Immunize.org, The Public Good Projects, and Shots Heard Round the World. Organizations such as these have laid a very solid foundation upon which we need to stand and not reinvent strategies and resources for each season.

## Figures and Tables

**Table 1:** COVID-19 Cumulative hospitalization rates by age up until 30 April 2024 [10,11].

Age Group	COVID-19 Hospitalizations per 100,000 Population
0–4 years 6–<12 months 1–4 years	72.8 162.1 32.1
5–17 years 5–11 years 12–17 years	10.3 8.9 11.9
18–49 years 18–29 years 30–39 years 40–49 years	37.4 26.1 39.6 49.1
50–64 years	109.2
≥65 years 65–74 years ≥75 years	537.6 277.6 917.3

**Table 2:** COVID-19 or influenza hospitalization across different age groups (1 October 2022 and 21 March 2023) [30].

Age Group	COVID-19 N = 93,888	Influenza N = 20,561	Count Ratio of COVID-19 versus Influenza
0–5 years	706 *	564	1.3
6–17 years	1529 *	1260	1.2
18–49 years	26,242 *	4693	5.6
50–64 years	22,947 *	5529	4.2
≥65 years	42,464 *	8515	5

Adapted from Kopel et al. [23]. * *p*-value < 0.001, COVID-19 vs. influenza.

**Table 3:** 2023/2024 COVID-19 and influenza vaccine uptake across age categories, as of March 2024.

	6 m–4 y	5–11 y	12–17 y	18–49 y	50–64 y	≥65 y
Influenza vaccine uptake	59.8%	52.3%	47.0%	36.8%	51.2%	73.7%
COVID-19 vaccine uptake	5.6%	12.6%	17.8%	13.6%	25.3%	42.5%

**Table 4:** 2023/2024 COVID-19 vaccine uptake across race/ethnicity in adults aged 18+ years, as of April 2024.

American Indian	Pacific Islander	Asian	Black	Hispanic	Multiple or Other Races	White, Non-Hispanic
15.6%	17.9%	21.8%	19.6%	15.6%	17.0%	25.5%

**Table 5:** Examples of presumptive offerings for COVID-19 vaccines.

“I see that you haven’t had your latest COVID-19 shot. This updated vaccine is recommended for you because your age increases your risk. I will have the nurse give it to you before you leave.” “It is that time of year again, you need to get your COVID-19 shot today. Let’s start with it.” “You mentioned you haven’t received the latest COVID-19 vaccine. Let’s get that shot in today so you are up to date.” “While you are here for your flu shot, we plan to give you the COVID-19 vaccine at the same time in your other arm. This will save you a trip and make sure you have the recommended vaccines for the fall and winter season.”

**Table 6:** Key takeaways and recommendations.

COVID-19 is the leading cause of respiratory infectious disease leading to hospitalizations and deaths regardless of age and comorbidities, both during and between seasonal peaks. Healthcare providers need to inform and educate their patients or the patient’s parents/caregivers on the disease burden and their risk for COVID-19-related morbidity and mortality. The SARS-CoV-2 virus mutates rapidly, measured over weeks and months rather than years. This explains the need for the COVID-19 vaccine to be updated regularly to ensure it covers the predominant circulating variants. Healthcare providers should inform their patients or the patient’s parents/caregivers of the need to remain up to date with the most recent COVID-19 vaccine to maintain ongoing protection. COVID-19 mRNA vaccines have demonstrated consistent clinical protection, significantly reducing the risk of hospitalization and death. Healthcare providers should presumptively recommend COVID-19 vaccination to all their patients or the patient’s parents/caregivers at the point-of-care (in all clinical settings and in retail pharmacies). For the upcoming 2024 fall season, COVID-19 vaccines will be licensed, recommended, and available at the point-of-care by the start of the respiratory vaccination campaign. HCPs should be co-administering COVID-19 vaccines with Influenza vaccines at the same visit to improve vaccination acceptance. Patients recognize their healthcare providers as the most trusted source of healthcare information. Delivering a strong, clear presumptive COVID-19 vaccine recommendation at the point-of-care (clinic setting or the pharmacy) has consistently been shown to increase vaccination acceptance whilst reducing the time needed with the patient. Providers should presumptively recommend the COVID-19 vaccine to every person 6 months of age and older at every opportunity. The misinformation that drives online conversations day after day is the misinformation that healthcare providers will hear from their patients when delivering a vaccination recommendation. Awareness of these trending topics and themes is critical for healthcare providers to optimally inform and educate their patients. Providers should regularly consult an infodemiology dashboard for awareness of the trending issues and how to categorize them within the distinct themes of perceived risk, safety, or vaccine effectiveness to be able to effectively provide evidence-based reassurance to their patients. Healthcare Providers, from their schooling through their residencies and into their professional careers, need to be trained in Communication skills to be able to better make clear recommendations and learn how to better communicate with patients/families with questions and/or concerns.
